# Case report: Thyroid sarcoidosis as a rare localization of the disease: Report of two cases and review of the literature

**DOI:** 10.3389/fmed.2023.1046420

**Published:** 2023-03-10

**Authors:** Svetlana Kašiković Lečić, Jovan Javorac, Aleksandra Lovrenski, Jelena Đokić, Danica Sazdanić Velikić, Dejan Živanović

**Affiliations:** ^1^Department of Internal Medicine, Faculty of Medicine, University of Novi Sad, Novi Sad, Serbia; ^2^Institute for Pulmonary Diseases of Vojvodina, Sremska Kamenica, Serbia; ^3^Department of Pathology, Faculty of Medicine, University of Novi Sad, Novi Sad, Serbia; ^4^Department of Geriatrics, Faculty of Medicine, University of Novi Sad, Novi Sad, Serbia; ^5^Department of Psychology, College of Social Work, Belgrade, Serbia; ^6^Department of Medical Sciences, College of Vocational Studies “Sirmium”, Sremska Mitrovica, Serbia

**Keywords:** sarcoidosis, thyroid, prevalence, diagnosis, treatment

## Abstract

Sarcoidosis is a multi-systemic disease of unknown etiology that is characterized by the formation of non-necrotizing and non-caseating granulomas in affected organs. Sarcoidosis granulomas can form in any organ, but the lungs and intrathoracic lymph nodes are the most commonly affected. Thyroid involvement in sarcoidosis is very rare, with prevalence estimates of 1–4.5% in case series of autopsied patients with systemic sarcoidosis. It is extremely rare for sarcoidosis to occur solely in the thyroid gland, but it is usually associated with the involvement of other organs, primarily the lungs and lymph nodes. Typical manifestations are diffuse goiter and solitary or multiple thyroid nodules. Thyroid function remains intact in the majority of cases, but sometimes it can result in hypothyroidism or hyperthyroidism. The diagnosis can be made after fine needle aspiration cytology, but usually it is diagnosed as an incidental finding while analyzing thyroidectomy tissue or during autopsy. Oral steroids are the cornerstone of thyroid sarcoidosis management, along with specific endocrinological treatment on some occasions. Given that we found only 71 reports of thyroid sarcoidosis available in the literature, we created and analyzed a cohort of 24 patients with thyroid sarcoidosis who were described in the literature in the 21st century and reported two additional cases of thyroid sarcoidosis.

## 1. Introduction

Sarcoidosis is a chronic systemic disease of an unknown etiology that is pathologically characterized by the formation of non-caseating and non-necrotizing epithelioid cell granulomas in affected organs or tissues (1). The proposed pathophysiologic mechanism involves an unidentified, most likely infectious antigen that causes an autoimmune reaction in genetically predisposed individuals (2). Sarcoidosis typically affects patients in their 40s and 50s, with women being slightly more affected, particularly in Afro-Americans, where the female-to-male ratio is 2:1 (3).

Sarcoidosis granulomas can develop in any organ, but the lungs and intrathoracic lymph nodes are the most commonly affected (around 90% of patients) (4). Other frequently involved organs include skin (around 50% of cases), liver (up to 80% in autopsy series reports), eyes (10–50%), spleen (6–16%) and peripheral lymph nodes (about 30%). Cardiac (3–39%) and neurosarcoidosis (3–10%) are uncommon but potentially fatal manifestations of sarcoidosis (5). In only 8% of cases, extrapulmonary sarcoidosis is not accompanied by pulmonary sarcoidosis (6), so it is expected that the majority of patients will have some type of pulmonary involvement during the course of the disease.

Involvement of the thyroid gland by sarcoidosis is very rare. In post-mortem studies of patients with previously diagnosed systemic sarcoidosis, the thyroid gland was affected in up to 4.5% (7). Usually, the extrathyroidal manifestation of the disease precedes the diagnosis of thyroid involvement. Some rare cases of sarcoidosis limited to the thyroid gland can also be found in the literature (8).

The aim of this paper was to conduct a review of the literature on the subject as well as to report two additional cases of our patients with thyroid sarcoidosis.

## 2. Case reports

### 2.1. Case report 1

A 53-year-old Caucasian female was admitted to a referral pulmonary institute after the chest X-ray showed infiltrations in the lungs and enlarged hilus on both sides. The chest X-ray was performed as a preoperative assessment for the scheduled thyroidectomy due to the euthyroid nodular goiter. The patient experienced a gradual enlargement of the thyroid over the past 4–5 months that caused difficulties with breathing and swallowing. Among the other complaints, she cited a dry cough in the last 2 months. She started treatment with antihypertensives 2 months earlier, while denying having any other comorbid conditions. The patient reported a positive family history of malignant lung and cardiovascular diseases, while her psycho-social history was unremarkable. Physical examination revealed an enlarged right lobe of the thyroid gland, while other findings were not abnormal.

**Figure 1:**
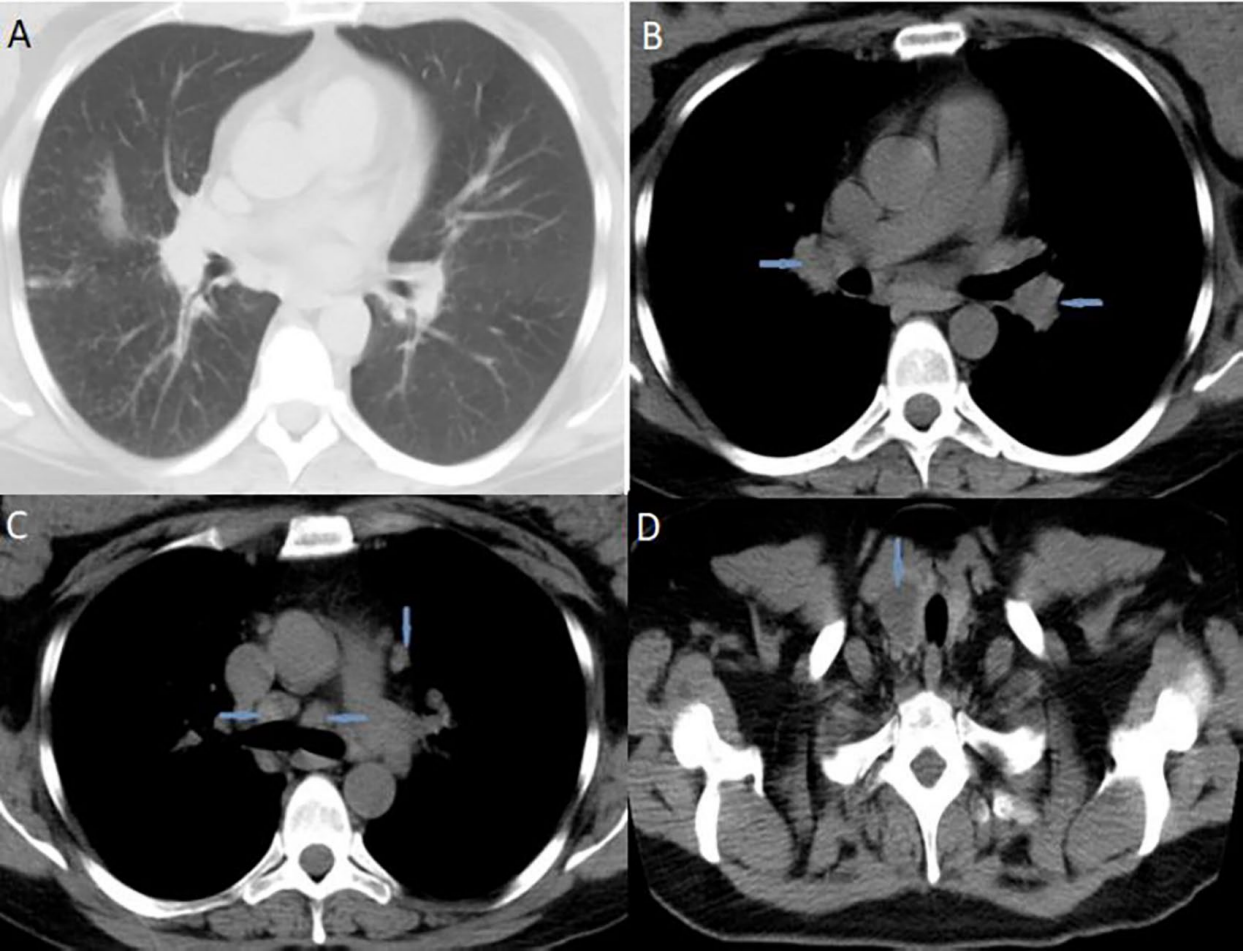
High-resolution computed tomography (HRCT) of the chest suggested pulmonary sarcoidosis: **(A)** micronodular lesions in pulmonary parenchyma; **(B)** hilar lymphadenopathy (blue arrows); **(C)** mediastinal lymphadenopathy (blue arrows). CT scans of the neck **(D)** indicated nodular mass inside the right thyroid lobe (blue arrow). HRCT, high-resolution computed tomography; CT, computed tomography.

**Figure 2:**
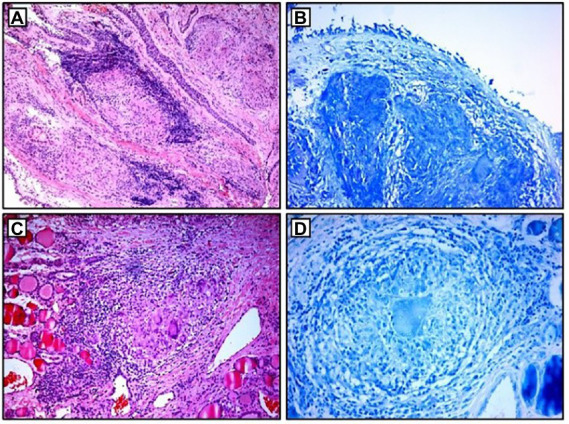
Histopathological examination: **(A,B)** non-necrotizing granulomas in bronchial mucosa and acid-fast stain showing absence of AFB; **(C,D)** thyroid tissue with non-necrotizing granulomas and acid-fast stain showing the absence of AFB. AFB, acid-fast bacilli.

**Figure 3:**
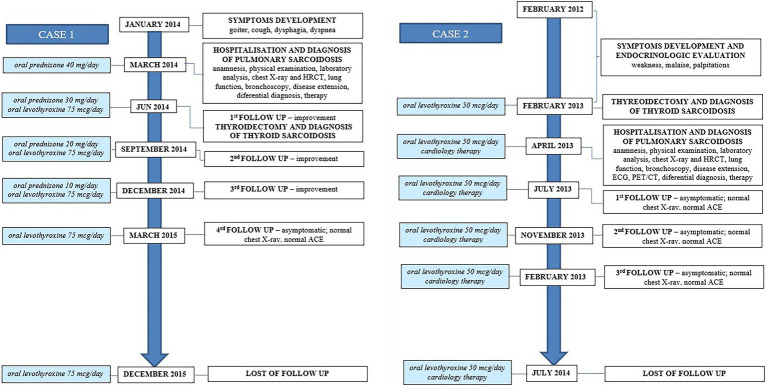
Case reports timeline. Presented according to CARE guidelines.

Blood laboratory analysis at the admission revealed elevated values of serum angiotensin-converting enzyme (ACE) and serum calcium, while other biochemical parameters, including the value of calcium in 24-h urine, were within reference range (Table 1). High-resolution computed tomography (HRCT) of the chest revealed numerous bilateral micronodular lesions in the pulmonary parenchyma and mediastinal and hilar lymphadenopathy (Figures 1A–C). An inhomogeneous, predominantly hypodense abnormality in the enlarged right thyroid lobe, 30×28 mm in diameter, was also observed (Figure 1D). No nodular changes were visible inside the left lobe. Pulmonary function tests and gas exchange during rest and exercise were both preserved. A bronchoscopy was performed. Histopathological examination confirmed non-necrotizing granulomatous inflammation of pulmonary parenchyma and lymph glands (Figures 2A,B). The ultrasound examination of the upper abdomen and the ophthalmological examination did not find any evidence of sarcoidosis extension. Tuberculosis was excluded since acid-fast staining of the patient’s sputum and lung biopsies revealed no acid-fast bacilli, while cultures on Mycobacterium tuberculosis and other mycobacteria later yielded negative results. Other causes of granulomatous inflammation, such as fungal infection, were excluded as well, since sputum and biopsies’ fungal cultures grew negative, while pathohistological staining of lung biopsies was negative for fungal infection. Following that, steroid therapy was administered, with a 40 mg/day (0.5 mg/kg) starting dose of oral prednisone. After 3 months of therapy, there was a radiological improvement on patient’s chest X-ray, with ACE levels in the reference range, while only minor changes in the size of the thyroid gland were observed. Considering that, a video-assisted lobectomy of the right thyroid lobe was performed shortly after. Histopathological examination of the thyroid tissue revealed non-necrotizing granulomas consistent with sarcoidosis (Figures 2C,D). Substitution therapy with 75 mcg/day oral levothyroxine was prescribed, along with the slow dose tapering of oral steroids over the next 9 months, when oral steroids were stopped (Figure 3). After that, the patient was asymptomatic, without visible changes on chest X-ray and no biochemical evidence of the disease’s activity. After being attentively monitored for the following 12 months, the patient stopped showing up for her scheduled checkups.

**Table 1:** Main socio-demographic, clinical and laboratory data from two reported patients.

Variables	Case report 1 (on admission)	Case report 2 (on admission)	Reference range
Gender	Female	Female	/
Age	53	68	/
Clinical manifestations	Goiter, dyspnea, dry cough, dysphagia	Weakness, malaise, palpitations	/
Organ involvement	Thyroid, lung, intrathoracic lymph nodes	Thyroid, lung, intrathoracic lymph nodes	/
Laboratory findings			
White blood cells (/L)	4.60 × 10^9^	5.20 × 10^9^	4–10 × 10^9^
Lymphocytes (/L)	1.20 × 10^9^	1.60 × 10^9^	1.19–3.35 × 10^9^
Neutrophils (/L)	3.24 × 10^9^	3.40 × 10^9^	2.06–6.49 × 10^9^
Hemoglobin (g/L)	137	126	120–170
Platelets (/L)	284 × 10^9^	275 × 10^9^	150–400 × 10^9^
Urea (mmol/L)	3.80	4.7	3–8
Creatinine (mmol/L)	82	80	50–100
AST (μkat/L)	0.34	0.28	0.0–0.5
ALT (μkat/L)	0.32	0.23	0.00–0.55
LDH (μkat/L)	5.49	5.65	3.8–7.6
CRP (mg/L)	1.73	4.25	0–5
aPTT (s)	30.1	Analysis not done	22–42
PT (%)	101	97	>70
Fibrinogen (g/L)	3.2	3.1	2.2–4.9
Serum ACE (nkat/L)	1,578	1808	200–1,133
Serum calcium (mmol/L)	2.73	2.2	2.15–2.55
24-h calcium (mmol/dU)	5.7	2.6	2.5–7.5
fT3 (pmol/L)	Analysis not done	12.2	1.5–4.1
fT4 (pg/mL)	16.0	42.6	10.2–24.5
TSH (mU/L)	0.65	0.07	0.3–4.2
TPO-Ab (U/ml)	14	540	<34
TG-Ab (U/ml)	9	290	<40
TR-Ab (U/L)	0.95	12	<1.5
Diagnosis	Bronchoscopy → thyroidectomy	Thyroidectomy → bronchoscopy	/
Treatment	Oral prednisone 40 mg/day with slow dose tapering over 12 months; Oral levothyroxine 75 mcg/day	Oral levothyroxine 50 mcg/day	/
Follow up	24 months; no symptoms, normal chest X-ray, reference level serum ACE	15 months; no symptoms, normal chest X-ray, reference level serum ACE	/

AST, aspartate aminotransferase; ALT, alanine aminotransferase; LDH, lactate dehydrogenase; CRP, C reactive protein; aPTT, activated partial thromboplastin time; PT, prothrombin time; ACE, angiotensin converting enzyme; fT3, free triiodothyronine; fT4, free thyroxine; TSH, thyroid-stimulating hormone; TPO-Ab, thyroid peroxidase antibodies; TG-Ab, thyroglobulin antibodies; TR-Ab, thyroid-stimulating-receptor antibodies.

### 2.2. Case report 2

**Figure 4:**
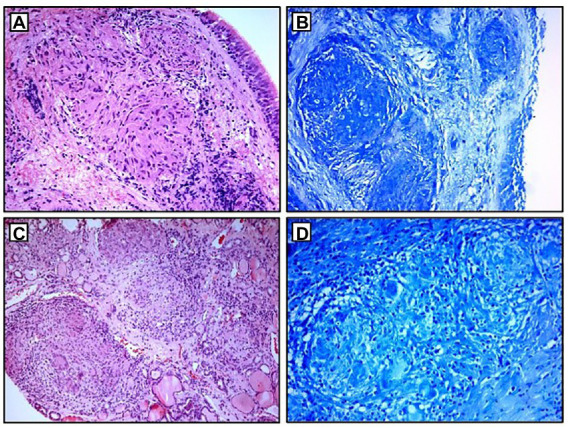
Histopathological examination: **(A,B)** non-necrotizing granulomas in bronchial mucosa and acid-fast stain showing absence of AFB; **(C,D)** thyroid tissue with non-necrotizing granulomas and acid-fast stain showing the absence of AFB. AFB, acid-fast bacilli.

A 68-year-old Caucasian female was admitted to a referral pulmonary institute for additional testing for suspected sarcoidosis. She had been treated with drugs for hyperthyroidism for about 35 years. However, around 12 months prior to the admission, she developed weakness, malaise, and a feeling of heart palpitations. An endocrinologist evaluated the patient multiple times. Since the hormone values (T3, T4, and TSH) were still irregular even after the prescribed therapy, she was treated surgically three months before the hospitalization. A total thyroidectomy was performed and definitive pathohistological findings revealed a polynodous goiter with numerous diffuse noncaseating granulomas whose morphology, number, and localization were typical of thyroid sarcoidosis (Figures 4C,D). Once the diagnosis of thyroid sarcoidosis was suspected, the patient was referred to a pulmonologist for further evaluation. At admission, she denied having any other comorbidity except thyroidectomy-associated hypothyroidism, which was managed with oral levothyroxine 50 mcg daily. She reported a positive family history of pulmonary tuberculosis. The psycho-social history as well as the physical examination were unremarkable.

Blood laboratory findings at the admission revealed elevated values of serum ACE, while other biochemical parameters, including the levels of serum calcium and calcium in 24-h urine, were within reference range (Table 1). Chest X-ray and HRCT scans were without lesions in the lung parenchyma and lymphadenopathy. Pulmonary function tests and gas exchange at rest and during exertion were preserved. Histopathological examination of transbronchial and bronchial biopsies revealed non-necrotizing granulomas consistent with sarcoidosis (Figures 4A,B). The ultrasound examination of the upper abdomen as well as the ophthalmological examination did not reveal any sarcoidosis extension. Considering family history, a thorough assessment of pulmonary tuberculosis was done, and it was excluded since acid-fast staining of the patient’s sputum and lung biopsies showed the absence of acid-fast bacilli, while cultures of Mycobacterium tuberculosis and other mycobacteria later on grew negative. The patient also had a negative result on the QuantiFERON TB-Gold In-Tube test. Other causes of granulomatous inflammation, such as fungal infection, were excluded as well, since fungal cultures of sputum and lung biopsies grew negative, while staining of lung biopsies was negative for fungal infection. During the cardiac evaluation, a heart rhythm disorder was recorded (left bundle branch block and first-degree atrioventricular block). Positron emission tomography/computed tomography was performed due to the suspicion of cardiac sarcoidosis; however, only bilateral hilar accumulation of radiopharmaceutical was observed. Once this diagnostic algorithm was finished, we decided not to administer steroids treatment, and the patient was discharged with thyroid substitution therapy (oral levothyroxine 50 mcg per day), while the cardiologist recommended conservative therapeutic approach of heart rhythm disorder. At the follow-up pulmonology visit 3 months later, the patient denied having any respiratory symptoms; the chest X-ray revealed no pathological alternations; and the serum ACE levels were now within the reference range. Without changing the treatment, she was followed every 3 months for the next 12 months until she stopped coming to scheduled controls.

### 2.3. Patients’ perspective

Patients agreed to our treatment plan and were thankful for the assistance provided. Since the patients stopped visiting our pulmonary institute after a certain period of follow-up, we contacted them by phone at the time of writing the study and received information that both patients are being monitored by endocrinologists, and periodically have check-ups with local pulmonologists, without symptoms characteristic of sarcoidosis.

## 3. Discussion

Thyroid involvement in sarcoidosis is very rare, with literature data estimating its prevalence of 1–4% in case series of autopsied patients with systemic sarcoidosis (9), while it is extremely rarely diagnosed *in vivo* (2). Thyroid sarcoidosis most commonly manifests clinically as gradual enlargement of the gland, which can be asymptomatic, cause pain in the gland, or cause compressive symptoms such as difficulty breathing and swallowing (9). The most frequently reported constitutional symptom is fatigue (10). Physical examination may reveal a diffusely and homogeneously enlarged thyroid, multinodular goiter, or cold solitary thyroid nodules, with or without cervical lymphadenopathy (9, 11).

Sarcoidosis is a disease that has been previously associated with the development of a variety of autoimmune and non-autoimmune endocrine disorders (12). Even though the majority of patients with sarcoidosis do not have an associated functional thyroid disorder (10), in about 2–20% of cases, sarcoidosis may be associated with either hypothyroidism or hyperthyroidism (2, 13). A significantly higher prevalence of clinical hypothyroidism and Graves’ disease was found in female patients (14). Similarities in the pathogenesis of Graves’ disease and sarcoidosis may explain the prevalence of thyroid autoimmunity in sarcoidosis patients, even when the thyroid is not involved (13). Simultaneous Graves’ disease and thyroid sarcoidosis may result in an unusual or suboptimal clinical response of Graves’ disease to various treatment modalities, such as antithyroid drug therapy and/or radioactive iodine therapy (7, 13). Hashimoto’s thyroiditis may also coexist with sarcoidosis, with several studies identifying it as the most common autoimmune disease associated with sarcoidosis (15). The association between Hashimoto’s thyroiditis and sarcoidosis could be due to increased thyroid-specific T-cell activation caused by increased expression of Th1/Th17 cells in both diseases (16). Non-necrotizing granulomas infiltration of the gland can also cause thyroid dysfunction and the development of hypothyroidism (7). Considering the above, clinicians should properly functionally evaluate certain sarcoidosis patients for thyroid disease, particularly determine thyroid antibodies for autoimmune thyroid diseases.

As with any other localization, the clinical manifestations of the disease, radiological findings, and histopathological evidence of non-caseating granulomas in the absence of any underlying microorganisms or particles must be considered for the diagnosis of thyroid sarcoidosis (17). Laboratory tests may be helpful and should begin with the measurement of serum thyroid hormones. The serum ACE, serum amyloid A (SAA), serum lysozyme, chitotriosidase, soluble interleukin-2 receptor (sIL2-R), and other biochemical markers may reflect the activity of sarcoidosis (18). Ultrasound is the preferred imaging modality for thyroid nodules. The typical ultrasonographic findings of thyroid sarcoidosis are scattered nodular shadows, 1–3 cm in diameter with irregular hypoechoic areas that reflect granuloma formation (8). Thyroid scintigraphy/radionuclide thyroid scan should be performed in patients presenting with a low serum TSH (19). The ultrasound-guided fine needle aspiration cytology (FNAc) is the preferred and the most commonly used method for evaluating thyroid changes seen on ultrasonography (20). This method has been shown to be very useful for detecting thyroid cancer, with high specificity and sensitivity. The false-negative rate of a benign interpretation is 0–3% (10). However, the diagnosis of thyroid sarcoidosis can be missed by FNAc, with findings indicating sarcoidosis malignancy or other non-sarcoid disorders (11). Potential explanations for this include the possibility of not obtaining a sample of granulomatous inflammation due to its uneven distribution in the gland, reactive follicular cells mimicking papillary structures, or misinterpreting condensed colloid as psammoma bodies (9, 11). Core needle biopsy (CNB) could be a useful diagnostic tool in cases of inconclusive FNAc results, with higher specificity and sensitivity than FNAc, avoiding misdiagnosis and overtreatment (9). In a retrospective study of nodules with two or more prior nondiagnostic FNAs, CNB was diagnostic in 86% compared with 29% for repeat FNAc (10). However, there are studies where CNB did not show superior diagnostic performance compared to FNAc for diagnosing thyroid nodules (21). In the majority of cases, thyroid sarcoidosis is diagnosed as an incidental finding through pathohistological examination of the surgically removed thyroid, which can be done due to glandular hyperplasia causing compressive symptoms, inability to regulate hormonal status despite therapy, due to previously suspected primary thyroid malignancy, or during autopsy (7).

The histopathological hallmark of non-caseating granulomas is non-specific to sarcoidosis. Due to similar clinical and ultrasonographic manifestations, the possibility of cervical lymph node involvement, and the low specificity of FNAc, the differential diagnosis between thyroid sarcoidosis and primary thyroid carcinoma is very challenging (9, 10). The possibility of coexistence of both thyroid sarcoidosis and thyroid carcinomas should also be contemplated (22). Although very rare, other granulomatous diseases should be considered when patients present with a neck mass and cervical lymphadenopathy, such as tuberculosis, atypical mycobacterial infections, brucellosis, fungal infections, subacute (DeQuervain’s) granulomatous thyroiditis, foreign body reactions, or non-Hodgkin’s lymphoma (7, 11, 23, 24).

Most patients with thyroid sarcoidosis do not require immunosuppressive therapy if the gland is not enlarged enough to cause compressive symptoms, if there are no functional disorders, or if there are no other organs affected (10). However, as isolated sarcoidosis of the thyroid is extremely rare (25), a large number of patients will present with multisystem sarcoidosis, necessitating the introduction of appropriate therapy. Systemic corticosteroids are the first-line treatment for sarcoidosis (26). Steroids have been shown to be effective in reducing the size of sarcoidosis masses in the thyroid (8), enough that surgical removal of the gland may be avoided in some cases. Second-line and third-line treatment, such as immunosuppressive and biological agents, should be considered for patients with corticosteroid-refractory disease, intolerable side effects, or corticosteroid toxicity (27). In cases of the presence of symptoms, especially if they are resistant to the applied therapy, or if there is a concern for malignancy, thyroidectomy is recommended (7), while the associated functional disorders are treated with appropriate therapy (10). The majority of nodules are asymptomatic, and with only 5 to 10% of nodules being malignant, the decision to operate is made on therapeutic or diagnostic grounds (28, 29). In addition, cases of spontaneous decreases in the size of thyroid granulomas have also been described in the literature (8).

The first report of thyroid sarcoidosis originates from an autopsy report in 1938 (30), with several case reports available after that. In a 2017 study by Okuma et al. (8), only 65 cases of systemic sarcoidosis with thyroid involvement were reported. Relying on that fact, using the selected key words “thyroid” and “sarcoidosis,” we reviewed the literature databases PubMed and Google Scholar over the next 6 years (January 1, 2017– June 1, 2022) and discovered an additional six case reports, bringing the total number of thyroid sarcoidosis reports in the available literature to 71. Due to the scarcity of data on this subject, we created a cohort of patients with diagnosed thyroid sarcoidosis who were described in the literature between January 1, 2000, and June 1, 2022. A total of 20 studies (2, 4, 7–11, 13, 17, 23, 25, 31–39), all of which were designed as case reports, and 24 patients with thyroid sarcoidosis were analyzed (Supplementary Table S1). Patients’ basic socio-demographic characteristics (gender, age), clinical manifestations of the disease, thyroid functional status, diagnostic procedure of thyroid sarcoidosis, possible extra-thyroid localizations of the disease, implemented treatment, and disease outcome were all collected.

Female patients were found to be the most represented in our sample, accounting for 83.3% of all patients. The sample’s average age was 50.2 years, ranging from 23 to 74. The majority of patients (45.8%) reported thyroid enlargement as the first clinical manifestation of the disease, as well as hyperthyroid symptoms and signs such as weight loss, heat intolerance, excessive sweating, flushing, palpitations, tachycardia, and tremors (in 29.2% of respondents). Constitutional symptoms like generalized weakness and malaise, as well as shortness of breath (dyspnea) and difficulty swallowing (dysphagia), were almost equally prevalent in our sample (20.8, 20.8, and 16.7%, respectively). The thyroid function analysis revealed that the majority of patients with thyroid sarcoidosis (63.6%) were euthyroid, 36.4% had laboratory signs of hyperthyroidism, and none had hypothyroidism. Antithyroid antibodies (thyroglobulin antibodies, thyroperoxidase, thyroid-stimulating-receptor antibodies) were reported for approximately half of the patients (13/24), and they were positive in 23% of cases. Initial thyroid ultrasound results were available for 17 of 24 patients. The majority of them (76.5%) presented with single or multiple thyroid nodules, with 46.1% also having a diffusely enlarged thyroid. In 11.7% of cases, heterogeneous thyroid tissue or a diffusely enlarged thyroid without nodules was found. In 14/24 patients, fine needle aspiration cytology (FNAc) was used as the initial diagnostic method to determine the etiology of ultrasound-observed pathological changes in the thyroid. Only 21.4% of cases had a FNAc finding of thyroid sarcoidosis, while in the rest of the cases it indicated primary thyroid malignancy (21.4%), inflammatory changes in the gland (14.3%), or other benign cytological characteristics of thyroid tissue (42.8%). A core needle biopsy (CNB) was performed on 3 of the 24 patients and was successful in detecting non-caseating granulomas each time (100%). However, in the majority of the patients (17/24), thyroid sarcoidosis was diagnosed following thyroidectomy. Only one patient (4.2%) had isolated thyroid sarcoidosis with no involvement of other organs. The remaining patients had multi-systemic sarcoidosis, with the lymph nodes of the chest (78.3%), lungs (47.8%), lymph nodes of the neck (34.8%), skin (17.4%), and liver (13%) being the most commonly affected. The majority of patients were treated endocrinologically with antithyroid drugs, radioactive iodine, or thyroidectomy, with oral steroids being used in 29.2% of cases. There was follow-up data available for 14/24 patients, with a mean follow-up of approximately 2 years and no progression of systemic sarcoidosis observed after treatment. The results of the analysis are also shown tabularly in Table 2.

**Table 2:** Literature review of patients with thyroid sarcoidosis.

	Number of patients	Percentage (%)
Gender		
Females	20/24	83.3
Males	4/24	16.7
Clinical manifestations		
Goiter	11/24	45.8
Hyperthyroid state	7/24	29.2
Constitutional symptoms	5/24	20.8
Dyspnea	5/24	20.8
Dysphagia	4/24	16.7
Thyroid function		
Euthyroid	14/22	63.6
Hyperthyroid	8/22	36.4
Hypothyroid	0/22	0
Ultrasound presentation		
Single or multiple thyroid nodules	13/17	76.5
Heterogeneous thyroid or a diffusely enlarged thyroid	2/17	11.7
Diagnosis		
Fine needle aspiration cytology	Diagnostic 3/14	21.4
	Non-diagnostic 11/14	78.6
Core needle biopsy	Diagnostic 3/3	100
	Non-diagnostic 0/3	0
Thyroidectomy	17/24	70.8
Sarcoidosis localization		
Only thyroid	1/24	4.2
Thyroid + chest lymph nodes	18/23	78.3
Thyroid + lungs	11/23	47.8
Thyroid + neck lymph nodes	8/23	34.8
Thyroid + skin	4/23	17.4
Thyroid + liver	3/23	13
Treatment		
Oral steroids	7/24	29.2

## 4. Conclusion

In conclusion, sarcoidosis involving the thyroid gland is a rare entity and may pose a diagnostic challenge. In the vast majority of cases, sarcoid involvement of the thyroid remains clinically insignificant. In clinically significant thyroid sarcoidosis, abnormalities in respect to gland morphology (i.e., nodules, goiter, thyroiditis) and function (hypo- and hyperthyroidism) may be isolated or combined. In patients with suspected thyroid sarcoidosis, thorough diagnosis and observation are necessary. The diagnosis may only be confirmed if known causes of granulomas and, in particular, sarcoid reactions are excluded. Often, particularly due to the diagnostic difficulties, surgery is indicated.

## Data availability statement

The original contributions presented in the study are included in the article/Supplementary material, further inquiries can be directed to the corresponding author.

## Ethics statement

Written informed consent was obtained from the individual(s) for the publication of any potentially identifiable images or data included in this article.

## Author contributions

SKL and JJ drafted the case report and performed the review of the literature. AL obtained the histopathological figures and descriptions. JĐ obtained the radiological figures and descriptions. DSV and DŽ contributed to the revising of the manuscript and language modifying. All authors contributed to the article and approved the submitted version.

## Conflict of interest

The authors declare that the research was conducted in the absence of any commercial or financial relationships that could be construed as a potential conflict of interest.

## Publisher’s note

All claims expressed in this article are solely those of the authors and do not necessarily represent those of their affiliated organizations, or those of the publisher, the editors and the reviewers. Any product that may be evaluated in this article, or claim that may be made by its manufacturer, is not guaranteed or endorsed by the publisher.

## Abbreviations

ACE: angiotensin-converting enzyme
TSH: thyroid-stimulating hormone
T4: thyroxine
T3: triiodothyronine
fT4: free thyroxine
fT3: free triiodothyronine
CNB: core needle biopsy
FNAc: fine needle aspiration cytology
FNAB: fine needle aspiration biopsy
US: ultrasonography
CT: computed tomography
HRCT: high-resolution computed tomography
LN: lymph nodes
TG-Ab: thyroglobulin antibodies
TPO-Ab: thyroperoxidase
TS-Ab: TSH-receptor antibodies
