# Supplementary material for: Case report: Thyroid sarcoidosis as a rare localization of the disease: Report of two cases and review of the literature
Source: Front Med (Lausanne). 2023 Mar 10;10:1046420. doi: 10.3389/fmed.2023.1046420 (PMC10036575; doi:10.3389/fmed.2023.1046420)
Supplement: Supplementary file 1 [file Table_1.DOCX]

Supplementary Material

**Supplementary Table 1.** Studies from the 21^st^ century where patients with thyroid sarcoidois were reported and clinical characteristics of these patients

| **Study** | **Age** | **Gender** | **Main complaints** | **Thyroid function** | **Thyroid antibodies** | **Thyroid imaging** | **Diagnosis of sarcoidosis** | **Extrathyroidal localizations** | **Treatment** | **Outcome** |
| --- | --- | --- | --- | --- | --- | --- | --- | --- | --- | --- |
| **Querternous et al.**  2021  (1) | 64 | female | syncope, memory loss, functional decline | low TSH, high T4, fT4 | negative | not reported | CNB – non-caseating granulomas | LN in the neck and chest;  central nervous system | antithyroid (methimazole) + oral steroids (prednisone) | after 6 weeks - improvement |
| **Katsamakas et al.**  2021  (9) | 70 | female | fatigue, malaise | normal TSH, fT4 | negative | US - 1 hypoechoic nodule of the left thyroid lobe | FNAc – suspected malignancy;  thyroidectomy pathology - non-caseating granulomas, no malignancy | LN in the neck and chest; lungs | thyroidectomy + levothyroxine | after 2 years – stable, no need for treatment of pulmonary sarcoidosis |
| **Katsamakas et al.**  2021  (9) | 74 | female | not reported | normal TSH, fT4 | negative | not reported | thyroidectomy pathology – non-caseating granulomas + papillary microcarcinoma | LN in the neck and chest; lungs | thyroidectomy + postoperative radioiodine ablation therapy | after 3 years – no sign of metastasis, stationary pulmonary sarcoidosis |
| **Bangolo et al.**  2021  (10) | 58 | female | cough, dyspnea, globus sensation, abdominal pain, constipation | normal TSH | negative | CT – 1 nodule of the left thyroid lobe | FNAc – benign;  CNB – granulomatous infiltration of the gland | LN in the chest; liver | thyroidectomy | not reported |
| **Okuma et al.**  2017  (3) | 66 | female | malaise, blurred vision | low TSH, normal fT3 and fT4 | negative | US – 1 hypoechoic nodule;  CT – low absorption mass in the right thyroid lobe | CNB – non-caseating epithelioid cell granulomas and lymphocytic infiltration | LN in the chest | no medication | after 57 weeks – thyroid nodule decreased in size, asymptomatic |
| **Wadhwa et al.**  2017  (30) | 62 | female | weight loss, abdominal pain, unconscious state | not reported | not reported | not reported | autopsy pathology – sarcoid granulomas | liver; spleen; lungs; abdominal LN | no medication (autopsy report) | not applicable |
| **Alsahwi et al.**  2016  (17) | 36 | female | weakness, dizziness, headaches, polyuria, polydipsia, dysphagia, constipation, depression, secondary amenorrhea | low TSH, normal fT3 and fT4 | not reported | US – 3 solid isoechoic nodules of the right thyroid lobe; | FNAc -granuloma formation | LN in the neck and chest; sellar/suprasellar mass | oral steroids (prednisone) + levothyroxine | after 6 months – decreased size of sellar/suprasellar mass, symptoms improvement, central endocrine disbalance remained |
| **Yanamandra et al.**  2013  (13) | 37 | male | weight loss, heat intolerance, excessive sweating, palpitations | low TSH, high T3 and T4 | negative | US – both lobes diffusely enlarged | FNAc – ill-formed elements of granulomatous tissue | LN of the chest; lungs | Antithyroid → radioiodine ablation therapy + oral steroids (prednisolone) | after 1 year - euthyroid |
| **Kmieć et al.**  2012  (5) | 52 | female | painful goiter, dysphagia, dyspnea | normal TSH and fT4 | not reported | US – heterogenous thyroid tissue with altered echogenicity | FNAc – histiocyte infiltration, multinuclear cells;  strumectomy pathology – sarcoid granulomas | LN in the chest; skin | oral steroids (ineffective) → partial thyroidectomy + levothyroxine | after 2 years – thyroid sarcoidosis recurrence treated with re-strumectomy;  after 4 years – in remission |
| **Kmieć et al.**  2012  (5) | 49 | female | painful goiter | normal TSH and fT4 | not reported | US – enlarged thyroid with multiple focal lesions | FNAc – granulomatous disease in the scar tissue area; strumectomy pathology – sarcoid granulomas + papillary cancer | LN in the neck and chest; lungs; skin | thyroidectomy + levothyroxine | after 2 years – in remission |
| **Manchanda et al.**  2013  (2) | 54 | female | goiter, dysphagia | not reported | not reported | US – thyromegaly with multiple isoechoic bilateral nodules;  CT – enlarged thyroid | FNAc – benign thyroid nodule;  thyroidectomy pathology (after 2 years) - non-caseating granulomas | LN in the chest | thyroidectomy + levothyroxine | not reported |
| **Hoang et al.**  2011  (11) | 65 | female | pressure and dyspnea in the supine position | normal TSH and fT4 | High TG-Ab, normal TPO-Ab | US – enlarged multinodular goiter | FNAc – benign adenomatoid goiter;  thyroidectomy pathology – numerous diffuse non-caseating granulomas | LN in the chest; lungs | thyroidectomy + levothyroxine | not reported |
| **Balasanthiran et al.**  2010  (31) | 70 | female | weight loss, constipation, abdominal pain | normal | not reported | not reported | partial thyroidectomy pathology – granulomatous thyroiditis | parathyroid; lungs | steroids | not reported |
| **Rodriguez et al.**  2007  (32) | 23 | female | goiter, symptoms of thyrotoxicosis | Low TSH, high T3 and T4 | not reported | not reported | thyroidectomy pathology – non-caseating granulomas | LN of the neck and chest | antithyroids → radioiodine ablation therapy → thyroidectomy | not reported |
| **Bruins et al.**  2007  (33) | 35 | female | goiter | normal TSH and fT4 | not reported | US – nodule of the left lobule | FNAc – suspected papillary carcinoma;  thyroidectomy pathology – papillary carcinoma + sarcoid granulomas | skin; lungs | thyroidectomy + levothyroxine | not reported |
| **Cabibi et al.**  2006  (24) | 42 | female | goiter, dysphagia | normal TSH, fT3 and fT4 | negative | US – multinodular goiter | thyroidectomy pathology – mild goiter and multiple non-caseating granulomas | none | thyroidectomy + levothyroxine | after 3 years – no signs of sarcoidosis |
| **Papi et al.**  2006  (34) | 30 | male | goiter, weight loss, palpitations, tremors, proptosis → fever, arthralgias, erythema nodosum | low TSH, high fT3 and fT4 | positive TG-Ab, TPO-Ab and TS-Ab | US – diffusely hypoechoic goiter without discrete nodules; | thyroidectomy pathology – multiple non-caseating granulomas | LN in the chest; lungs | methimazole → propylthiouracil + oral steroids (prednisone) → near-total thyroidectomy | stable on antithyroids and low-dose steroids |
| **Papi et al.**  2006  (34) | 57 | female | generalized weakness | low TSH, high fT3 and fT4 | negative | US – huge multinodular goiter with large nodule of the left lobe | FNAc – hyperplastic cells without atypia;  near-total thyroidectomy pathology – multinodular goiter, several non-caseating granulomas | LN of the chest; lungs | methimazole + methylprednisolone → near-total thyroidectomy | after 1 year – stable and euthyroid on levothyroxine and low-dose methylprednisolone |
| **Ozkan et al.**  2005  (22) | 42 | female | palpitation, flushing, goiter | normal TSH and fT3 | not reported | US – multiple, solid nodules in the remaining tissue (23 years before strumectomy was made) | FNAc – benign fibrosis configurations;  thyroidectomy pathology – non-caseating granulomas | liver | total thyroidectomy | after 3 years – uneventful |
| **Ozkan et al.**  2005  (22) | 53 | female | weight loss, tachycardia, fatigue, heat intolerance | normal TSH and fT3 | not reported | US – multiple hypoechoic solid nodules | FNAc – benign colloidal accumulations;  thyroidectomy pathology – non-caseating granulomas | LN in the chest | total thyroidectomy | after 6 months – uneventful |
| **Gentilucci et al.**  2004  (35) | 59 | male | fever, productive cough, dyspnea, painful goiter | low TSH, high fT3 and fT4 | negative | CT – thyroid enlargement, nonhomogeneous nodule of the right lobe | thyroidectomy pathology – interstitial non-caseating granulomas | LN in the neck and chest; lungs; heart | total thyroidectomy | not reported |
| **Saydam et al.**  2003  (36) | 45 | female | hoarseness, goiter | normal TSH, fT3 and fT4 | negative | not reported | FNAc – suspected malignancy;  thyroidectomy pathology – noncaseous granulomatous reaction | skin | total thyroidectomy | after 7 months – no evidence of systemic sarcoidosis |
| **Yarman et al.**  2001  (37) | 28 | female | weight loss, tremor, palpitations, goiter | low TSH, high T3, T4 and fT4 | positive TG-Ab, TPO-Ab and TS-Ab | US – heterogenous thyroid parenchyma | FNAc – no sarcoid granulomas;  thyroidectomy pathology – non-caseating granulomas | LN of the chest; lungs | propylthiouracil + propranolol → total thyroidectomy + levothyroxine | after 4 months – no evidence of systemic sarcoidosis |
| **Zimmermann-Belsing et al**.  2000  (38) | 27 | male | weight loss, tremor, goiter, tachycardia | low TSH, high T3 and T4 | not reported | not reported | thyroidectomy pathology – sarcoidosis + papillary carcinoma + Graves’s disease | LN of the neck and chest; lungs | thiamazole + propranolol → total thyroidectomy → iodine therapy → levothyroxine | After 5 years – sarcoidosis of skin, thyroid-associated ophthalmology, no signs of malignancy |

**Legend**: TSH – thyroid-stimulating hormone, T4 - thyroxine, T3 -triiodothyronine fT4 – free thyroxine, fT3 – free triiodothyronine, CNB - core needle biopsy, FNAc - fine needle aspiration cytology, FNAB – fine needle aspiration biopsy, US – ultrasonography, CT – computed tomography, LN - lymph nodes, TG-Ab – thyroglobulin antibodies, TPO-Ab – thyroperoxidase, TS-Ab – TSH-receptor antibodies
